# Out-of-Hospital Perimortem Cesarean Section as Resuscitative Hysterotomy in Maternal Posttraumatic Cardiac Arrest

**DOI:** 10.1155/2014/121562

**Published:** 2014-10-30

**Authors:** Francesca Gatti, Marco Spagnoli, Simone Maria Zerbi, Dario Colombo, Mario Landriscina, Fulvio Kette

**Affiliations:** ^1^Anaesthesia and Intensive Care Unit 2, Sant'Anna Hospital, San Fermo della Battaglia, 22020 Como, Italy; ^2^Anaesthesia and Intensive Care Unit 1, Sant'Anna Hospital, San Fermo della Battaglia, 22020 Como, Italy; ^3^Bergamo 118 Operative Dispatch Center, Azienda Regionale Emergenza Urgenza (AREU), Via Campanini 6, 20124 Milan, Italy

## Abstract

The optimal treatment of a severe hemodynamic instability from shock to cardiac arrest in late term pregnant women is subject to ongoing studies. However, there is an increasing evidence that early “separation” between the mother and the foetus may increase the restoration of the hemodynamic status and, in the cardiac arrest setting, it may raise the likelihood of a return of spontaneous circulation (ROSC) in the mother. This treatment, called Perimortem Cesarean Section (PMCS), is now termed as Resuscitative Hysterotomy (RH) to better address the issue of an early Cesarean section (C-section). This strategy is in contrast with the traditional treatment of cardiac arrest characterized by the maintenance of cardiopulmonary resuscitation (CPR) maneuvers without any emergent surgical intervention. We report the case of a prehospital perimortem delivery by Caesarean (C) section of a foetus at 36 weeks of gestation after the mother's traumatic cardiac arrest. Despite the negative outcome of the mother, the choice of performing a RH seems to represent up to date the most appropriate intervention to improve the outcome in both mother and foetus.

## 1. Introduction

Traumatic cardiac arrest is a life threatening situation with a negligible survival rate [1–3].

A traumatic cardiac arrest in pregnant women is even more challenging condition due to physiological changes which may further limit the generation of an adequate cardiac output (CO). During manual chest compressions cardiac output achieves approximately 30% of the normal CO. In a late term pregnant woman this is further compromised because the aorto-caval compression by the gravidic uterus hampers the venous return and the CO generated by precordial compression is no greater than 10% [4–6].

CPR is often performed only via the standard maneuvers characterized by assisted ventilation and chest compression in left lateral tilt with the aid of a wedge (Cardiff wedge) or via a manual uterus displacement aimed to facilitate the venous return. The evidence of a temporal relationship between emptying the uterus in order to reduce the aorto-caval compression and the increase in CPR effectiveness has suggested that an early C-section may be the most appropriate intervention to improve the outcome in both mother and foetus. Katz and other authors recently pointed out the potential effectiveness of such emergent surgical intervention, although they also emphasized that there is still a cultural and psychological gap in managing correctly these situations [5, 6]. Lack of knowledge, skills and refusal of an aggressive and highly invasive treatment have been recognized as the main determinant of failed emergent C-section [5–9].

The more recent American Heart Association (AHA) and European Resuscitation Council (ERC) Guidelines on CPR provided recommendations on resuscitation in special circumstances such as traumatic cardiac arrest and pregnancy. These recommendations, however, are mainly based on reviews, anecdotal reports, and* in vitro* studies [4, 7].

According to the suggested strategy, we wish to report on a case of a traumatic cardiac arrest in which an emergent delivery was performed on the site of a car crash by the emergency physician while CPR was still ongoing.

## 2. Case Presentation

The 1-1-8 Emergency Medical Service (EMS) Operative Centre (O.C.) was activated on August 17, 2010, following a traffic accident involving two cars in a high-energy, head-on collision. The O.C. referred seven people involved, two of whom were apparently unconscious and trapped in their car.

EMS dispatched three ambulances with certified basic life support and defibrillation (BLSD) rescuers and one advanced life support (ALS) team. This is constituted by a physician, mainly an anaesthesiologist, by a registered nurse certified in out-of-hospital intervention, and by a car driver certified in BLSD maneuvers.

The first ambulance team confirmed cardiac arrest in a woman who was at the 36th gestational week and begun immediately CPR maneuvers. The ALS team arrived soon after and begun standard advanced resuscitation. The rhythm monitoring confirmed a pulseless electrical activity (PEA) with a heart rate (HR) of 30 beats per minute (bpm). Three milligrams (mg) atropine and eight mg epinephrine were administered intravenously. Oral tracheal intubation was successfully performed at the first attempt despite the presence of blood into the pharynx and the patient was manually ventilated at 1.0 inspired oxygen fraction (FiO_2_).

The clinical examination highlighted head trauma, a facial cyanosis, thoracic trauma with reduced left expansion in the setting of a global severe chest trauma, and an abdominal seat-belt hematoma. According to the clinical findings it was hypothesized that head and/or thoracic trauma could have been the most reliable causes of cardiac arrest. This led the physician to introduce a chest needle for an initial treatment of a potential hypertensive pneumothorax, but the maneuver was uneventful.

The suspect of an abdominal massive hemorrhage led also to the infusion of a volume of about two liters of crystalloids through a left subclavian cannulation. Unfortunately, the lack of ultrasound did not allow the possible recognition of such life threatening condition.

End-tidal CO_2_ monitoring was not performed because of the lack of the device.

Twelve minutes after cardiac arrest, the EMS physician decided to carry the patient on the spinal board into the ambulance and to proceed with a Perimortem Caesarean Section (PMCS), in agreement with the 1-1-8 O.C. physician and the gynaecologist on duty.

After a quick disinfection, a deep umbilical-pubic laparotomy was performed. There were no signs of bleeding. The uterus was gestational and cut with a lengthwise incision. The head of the foetus was free from the umbilical cord. There was no evidence of meconium.

A female foetus was extracted 3 minutes after onset of the procedure. At the first minute the Apgar score was 3 (the HR was 60 beats per minute, the breathing activity was absent, the muscular tone was flaccid, the corneal reflexes were absent, there was no grimace, and the skin was pink-coloured).

Newborn life support, consisting of airways aspiration and orotracheal intubation with uncuffed tube was performed. The baby was wiped, dried, and warmed up. An intraosseous needle was placed because of lack of vein cannulation.

The Apgar score at the 5th minute was 6, the HR was higher than 100 bpm, and the skin was pink-coloured; the reflexes were absent, there was no grimace, and there was spontaneous but irregular respiratory drive.

Maternal life support was continued by the nurse. After PMCS the hemodynamic condition did not change. Nevertheless, both mother and baby were carried to the hospital. The mother was declared deceased soon after arrival at the emergency department (ED) whereas the newborn was handed over by the neonatologist and admitted to the neonatal intensive care unit (NICU).

Subsequent anatomical findings revealed that the mother deceased because of amniotic pulmonary embolism associated with additional minor traumatic injuries. No major bleeding was identified.

At a distance of four years from the event the baby had a neurological development slightly reduced with a slower language acquisition and a normal physical growth.

## 3. Discussion

Historically a postmortem foetus extraction was described as early as 715 before Christ when the Roman King Numa Pompilius decreed that no child should be buried within his mother (for religious reasons) since some foetus was found still alive [5].

One of the first positive case report of PMCS was described by De Pace and coworkers in 1982 where a woman developed cardiac arrest (CA) during fiberoscopy. After 20 minutes of CPR, PMCS was performed and ROSC was soon achieved in the mother while the baby recovered uneventfully. Both had a full long term neurological survival [10].

The true incidence of cardiac arrest during pregnancy has been estimated to be about 1 over 30,000 pregnancies [11].

Despite the direct deaths in the UK account for less than 50% of the total maternal deaths, there are “indirect” deaths related to psychiatric disease which lead to attempted suicides during pregnancy. These deaths account for 20% of worldwide maternal deaths [12].

Although resuscitation algorithms during cardiac arrest are the same for pregnant patients as for non-pregnant patients, the anatomical and physiological modifications of the late term pregnant woman impose more aggressive interventions such as advanced airway management and the attention to left lateral tilt or lateral displacement of the uterus. The ALS guidelines point out the possible use of a PMCS as soon as a pregnant woman develops cardiac arrest [4, 7].

The striking decision to intervene with a PMCS relied on the four “gold points”: the stay and play strategy, the Katz's paradox, the four-minute rule, and the surgical techniques.

According to the stay and play strategy our decision was chosen by judging the difficulty to maintain adequate coronary and cerebral perfusion via manual chest compressions due to the lack of both a mechanical chest compressor and a Cardiff wedge [5, 13, 14]. We estimated that transportation would have dramatically worsened the overall hemodynamic conditions due to the poor perfusion generated by our chest compressions. Yet, the lack of the end-tidal CO_2_ monitoring impeded us to adequately detect the effectiveness of cardiac compressions. The further reduction of the already low cardiac output by the aorto-caval compression induced us to empty the uterus according to the Katz's principle. Accordingly, the uterus should be emptied and the baby delivered to achieve an improved hemodynamic status during both non-cardiac and cardiac arrest condition even when the foetus is dead in the attempt to save at least the mother [5]. This concept was based on previous physiopathological evidence by Kerr as far as in 1965 when the effects of vena cava compression and the reduction of venous return by the gravidic uterus were demonstrated [15].

As per the four-minute rule, emergency Cesarean section should be performed when the gravidic uterus is considered to be the cause of maternal hemodynamic impairment due to aorto-caval compression regardless of fetal viability [13, 15–19]. The “emergency hysterotomy” is recommended to be initiated within four minutes of maternal cardiopulmonary arrest if ALS maneuvers are unsuccessful, although this is a class IIb intervention with a level of evidence (LOE) C [4]. According to Bloomer, the term “resuscitative hysterotomy” should be preferred instead of Perimortem Cesarean Section as it better represents a way to reverse cardiac arrest rather than simply being a last chance of resuscitative efforts [20, 21].

The issue on the best timing within which the procedure should be performed is still debated. Indeed, it is widely recognized that emergency hysterotomy should be performed as early as possible preferentially after two cycles of CPR (after four minutes after cardiac arrest), without wasting time to perform ultrasonography or Doppler foetal heart sound examination [4, 22, 23].

The surgical technique may represent another option to speed up an emergency C-section. Under these conditions the preferred surgical laparotomy consists of a vertical umbilical-pubic incision which differs from the classical Pfannenstiel incision used in the in-hospital setting [5, 24]. Our surgical procedure was kept as simple as possible, considering the limited expertise of the emergency physician. In fact, although the experts suggest that the placenta should be removed from the uterus as well, we just left it in situ and packed the opened abdomen. Indeed Draycott and the TOSTI study demonstrated that introduction of obstetric emergencies training courses was associated with a significant increase of team work and reduction in low 5-minute Apgar scores and hypoxic-ischemic encephalopathy [6, 25].

In our case this intervention did not have positive effects on the mother but rather on the baby. The subsequent autopsy showed posttraumatic amniotic fluid embolism (AFE) [26] as the main cause of the death rather than massive abdominal hemorrhage. The deceleration of the mother's body inside the vehicle during the impact could be involved in the mechanism of injury, as demonstrated by Crosby and coworkers as far as 1968 [27]. The AFE was the likely reason why ROSC could not be achieved even though the C-section was performed on the scene within 20 minutes without maternal ALS interruption. This time frame was not a limitation since a complete neurological recovery after 30 minutes of CPR was documented in women who experienced cardiac arrest due to traumatic events [28].

Despite the positive outcome we recognized that this case has some limitations: lack of adequate ETCO_2_ monitoring and ultrasound, absence of circulatory supporting devices, and limited experience. Nevertheless our report represents an unusual case of emergency delivery performed in a mother victim of traumatic cardiac arrest in which the C-section led to a full neurological recovery of the baby at a distance of four years from the event.

By confirming the fortuity of our result, we wish to point out that the key to salvage a pregnant woman in cardiac arrest is to separate the maternal-foetal unit according to the Katz's principle and to emphasize what the AHA guidelines report: “*This strategy seems to be the best way to manage the serious impairment in pregnancy (>24 weeks). The hysterotomy also allows access to the infant so the newborn resuscitation can begin*” [4, 7].

## References

[1] Perkins G. D., Brace S., Gates S. (2010). Mechanical chest-compression devices: current and future roles. *Current Opinion in Critical Care*.

[2] Huber-Wagner S., Lefering R., Qvick M., Kay M. V., Paffrath T., Mutschler W., Kanz K.-G. (2007). Outcome in 757 severely injured patients with traumatic cardiorespiratory arrest. *Resuscitation*.

[3] Pickens J. J., Copass M. K., Bulger E. M. (2005). Trauma patients receiving CPR: predictors of survival. *Journal of Trauma: Injury Infection & Critical Care*.

[4] Hoek T. L. V., Morrison L. J., Shuster M., Donnino M., Sinz E., Lavonas E. J., Jeejeebhoy F. M., Gabrielli A. (2010). Part 12: cardiac arrest in special situations: 2010 American Heart Association guidelines for cardiopulmonary resuscitation and emergency cardiovascular care. *Circulation*.

[5] Katz V. L. (2012). Perimortem cesarean delivery: its role in maternal mortality. *Seminars in Perinatology*.

[6] Dijkman A., Huisman C. M. A., Smit M., Schutte J. M., Zwart J. J., Van Roosmalen J. J., Oepkes D. (2010). Cardiac arrest in pregnancy: increasing use of perimortem caesarean section due to emergency skills training?. *BJOG: An International Journal of Obstetrics & Gynaecology*.

[7] Soar J., Perkins G. D., Abbas G., Alfonzo A., Barelli A., Bierens J. J. L. M., Brugger H., Deakin C. D., Dunning J., Georgiou M., Handley A. J., Lockey D. J., Paal P., Sandroni C., Thies K.-C., Zideman D. A., Nolan J. P. (2010). European Resuscitation Council guidelines for resuscitation 2010 section 8. Cardiac arrest in special circumstances: electrolyte abnormalities, poisoning, drowning, accidental hypothermia, hyperthermia, asthma, anaphylaxis, cardiac surgery, trauma, pregnancy, electrocution. *Resuscitation*.

[8] Cohen S. E., Andes L. C., Carvalho B. (2008). Assessment of knowledge regarding cardiopulmonary resuscitation of pregnant women. *International Journal of Obstetric Anesthesia*.

[9] Draycott T., Sibanda T., Owen L., Akande V., Winter C., Reading S., Whitelaw A. (2006). Does training in obstetric emergencies improve neonatal outcome?. *BJOG: An International Journal of Obstetrics & Gynaecology*.

[10] de Pace N. L., Betesh J. S., Kotler M. N. (1982). “Postmortem” cesarean section with recovery of both mother and offspring. *The Journal of the American Medical Association*.

[11] Lewis G. (2004). CEMACH, Department of health, Welsh office, Scottish Office, Department of Health and social services, Northern Ireland, Why mothers die. Report on enquiries into maternal deaths in the United Kingdom. *Registered Charity no.*.

[12] Lewis G. (2007). *The Confidential Enquiry into Maternal and Child Health CEMACH. Saving Mothers’ Lives: Reviewing Maternal Deaths to Make Motherhood Safer 2003–2005. The Seventh Report on Confidential Enquirers into Maternal Deaths in the United Kingdom*.

[13] Katz V., Balderston K., Defreest M. (2005). Perimortem cesarean delivery: were our assumptions correct?. *American Journal of Obstetrics & Gynecology*.

[14] Ellington C., Katz V. L., Watson W. J., Spielman F. J. (1991). The effect of lateral tilt on maternal and fetal hemodynamic variables. *Obstetrics & Gynecology*.

[15] Kerr M. G. (1965). The mechanical effects of the gravid uterus in late pregnancy. *The Journal of obstetrics and gynaecology of the British Commonwealth*.

[16] Katz V. L., Dotters D. J., Droegemueller W. (1986). Perimortem cesarean delivery. *Obstetrics & Gynecology*.

[17] Bamber J. H., Dresner M. (2003). Aortocaval compression in pregnancy: the effect of changing the degree and direction of lateral tilt on maternal cardiac output. *Anesthesia & Analgesia*.

[18] Goodwin A. P. L., Pearce A. J. (1992). The human wedge: a manoeuvre to relieve aortocaval compression during resuscitation in late pregnancy. *Anaesthesia*.

[19] Marx G. F. (1982). Cardiopulmonary resuscitation of late-pregnant women. *Anesthesiology*.

[20] Katz V. L., Wells S. R., Kuller J. A., Hansen W. F., McMahon M. J., Bowes W. A. (1995). Cesarean delivery: a reconsideration of terminology. *Obstetrics & Gynecology*.

[21] Bloomer R., Reid C., Wheatley R. (2011). Prehospital resuscitative hysterotomy. *European Journal of Emergency Medicine*.

[22] Kue R., Coyle C., Vaughan E., Restuccia M. (2008). Perimortem Cesarean section in the helicopter EMS setting: a case report. *Air Medical Journal*.

[23] Brun P. M., Chenaitia H., Dejesus I., Bessereau J., Bonello L., Pierre B. (2013). Ultrasound to perimortem caesarean delivery in prehospital settings. *Injury*.

[24] Warraich Q., Esen U. (2009). Perimortem caesarean section. *Journal of Obstetrics and Gynaecology*.

[25] van de Ven J., Houterman S., Steinweg R. A. J. Q., Scherpbier A. J. J. A., Wijers W., Mol B. W. J., Oei S. G. (2010). Reducing errors in health care: cost-effectiveness of multidisciplinary team training in obstetric emergencies (TOSTI study); a randomised controlled trial. *BMC Pregnancy and Childbirth*.

[26] Thongrong C., Kasemsiri P., Hofmann J. P. (2013). Amniotic fluid embolism. *International Journal of Critical Illness and Injury Science*.

[27] Crosby W. M., Snyder R. G., Snow C. C., Hanson P. G. (1968). Impact injuries in pregnancy. I. Experimental studies. *The American Journal of Obstetrics and Gynecology*.

[28] Capobianco G., Balata A., Mannazzu M. C., Oggiano R., Pinna Nossai L., Cherchi P. L., Dessole S. (2008). Perimortem cesarean delivery 30 minutes after a laboring patient jumped from a fourth-floor window: baby survives and is normal at age 4 years. *The American Journal of Obstetrics and Gynecology*.

